# Self‐reported changes in adolescent mental health, deliberate self‐harm, substance use, and help‐seeking behavior before and after the COVID‐19 pandemic – A Finnish time‐trend study

**DOI:** 10.1111/camh.70040

**Published:** 2025-10-08

**Authors:** Andre Sourander, Xiao Zhang, Omid Dadras, Anne Abio, Kaisa Mishina, Tiia Ståhlberg, Yuko Mori, Sonja Gilbert, Emmi Heinonen, David Gyllenberg

**Affiliations:** ^1^ Research Centre for Child Psychiatry University of Turku Turku Finland; ^2^ Invest Research Flagship University of Turku Turku Finland; ^3^ Department of Child Psychiatry Turku University Hospital Turku Finland; ^4^ Department for Adolescent Psychiatry Turku University Hospital Turku Finland; ^5^ Department of Adolescent Psychiatry Helsinki University Hospital Helsinki Finland

**Keywords:** Adolescent, mental health, COVID‐19, deliberate self‐harm, substance use, help‐seeking behavior

## Abstract

**Background:**

Despite the impact that the COVID‐19 pandemic had on adolescents' mental health, there is a lack of studies comparing it pre‐ and postpandemic using consistent designs and measurements. Therefore, this study aimed to analyze changes in adolescent psychopathology, deliberate self‐harm behavior, substance use, and help‐seeking behavior pre‐ and post‐COVID‐19, with an identical study design.

**Methods:**

The study included three repeated cross‐sectional studies conducted in 2014, 2018, and 2023, including self‐reported data from Finnish secondary school students in grades 7 to 9, aged 13 to 16 (*n* = 9,024). The survey measured demographic information, mental health using the Strengths and Difficulties Questionnaire (SDQ), deliberate self‐harm behavior, substance use, and help‐seeking behavior.

**Results:**

Comparing data in 2023 with 2018, there were significant increases among females in total SDQ score (OR 2.1, 98.33% CI 1.7–2.7), conduct problems (OR 1.7, 98.33% CI 1.1–2.7), emotional symptoms (OR 1.8, 98.33% CI 1.5–2.3), and hyperactivity symptoms (OR 2.8, 98.33% CI 2.2–3.6). Perceived severe overall difficulties (OR 2.8, 98.33% CI 2.0–3.7), weekly smoking (OR 2.7, 98.33% CI 1.5–4.9), and seeking help (OR 1.5, 98.33% CI 1.2–2.0) increased. For males, increases were noted only in hyperactivity symptoms (OR 2.5, 98.33% CI 1.2–1.9) and perceived severe overall difficulties (OR 1.5, 98.33% CI 1.0–2.1), along with a decrease in alcohol consumption (OR 0.7, 98.33% CI 0.5–0.9). By contrast, the period from 2014 to 2018 showed minimal changes.

**Conclusion:**

The concerning rise in psychopathology after the COVID‐19 pandemic, particularly among females, highlights the importance of early detection and effective interventions.

**Key Practitioner Message**

**What is currently known?**

- Adolescents' mental health has shown a notable decline after the COVID‐19 pandemic.

**What has been shown?**

- This repeated cross‐sectional study revealed increased psychopathology, deliberate self‐harm, and help‐seeking behavior, with minimal changes in substance use in the Finnish context.

**What is the significance of this for clinical practice?**

- The increased difficulties call for gender‐sensitive interventions and accessible services to address rising help‐seeking behavior.

## Introduction

Several systematic reviews have demonstrated significant increases in adolescent mental health problems, particularly anxiety, depression, and deliberate self‐harm behavior following the COVID‐19 pandemic (Bower et al., 2023; Kauhanen et al., 2023; Orban et al., 2024; Sayed et al., 2024). These issues not only compromise individual well‐being but also carry significant societal implications, including increased healthcare costs and reduced productivity. Pandemic‐related factors, including fear of infection, concerns for loved ones, restrictions on social and physical activities due to lockdowns, isolation, family conflicts, and excessive exposure to media, are widely considered key contributors (Cheng, Wang, Wang, Zou, & Qu, 2023; Keyes & Platt, 2024; Panchal et al., 2021; Peng et al., 2023; Wiguna et al., 2024).

A recent review indicates increased internalizing problems among adolescents throughout the 21st century, attributed to earlier puberty onset, hormonal changes, increased use of digital devices, the COVID‐19 pandemic, and worsening macro‐economic conditions, such as economic instability and inequality (Keyes & Platt, 2024). There is also an expansion of adolescents reporting internalizing and several other problems over time, which needs to be considered when interpreting research findings (Haslam, 2016). Accurate assessment of time‐trend changes in adolescent mental health requires repeated population‐based cross‐sectional surveys with consistent sampling, regions, and methodologies (Collishaw, 2015). Many studies comparing prepandemic and early pandemic mental health face methodological inconsistencies, small samples, or a narrow focus on internalizing problems (e.g., Hafstad, Sætren, Wentzel‐Larsen, & Augusti, 2022; Ravens‐Sieberer et al., 2023).

Few population‐based studies have employed repeated cross‐sectional surveys with consistent methodologies to evaluate mental health changes before and after the pandemic, particularly in later phases (Kauhanen et al., 2023; Orban et al., 2024). A Norwegian study using nationwide school surveys from 2014 to 2022 observed increased adolescent cannabis and illicit drug use (Myhr, Vesterbekkmo, Samarawickrema, & Sund, 2024). An Icelandic study reported increases in depression and worsened mental well‐being from 2018 to 2022 (Thorisdottir et al., 2023). In Finland, a nationwide survey investigated generalized anxiety, depression, loneliness, social anxiety, and well‐being, reporting an increase in generalized anxiety, depression, and loneliness from 2019 to 2023, social anxiety from 2015 to 2023, along with a decline in overall well‐being from 2017 to 2023 (Kiviruusu et al., 2024). Another study from the Health Behavior in School‐aged Children examined Finnish adolescents between 2018 and 2022, identifying deterioration in mental health with increases in loneliness and psychosomatic complaints (Gustafsson et al., 2023). A German study spanning 2009 to 2022 examined subjective health and life satisfaction (Reiß et al., 2024). These studies compared multiple time points before and after the pandemic but focused primarily on internalizing problems, lacking consistent assessment of externalizing problems, substance use, and help‐seeking behavior. These gaps underscore the need for comprehensive studies using consistent methodologies to address these areas.

This study utilized three population‐based cross‐sectional samples from before the COVID‐19 pandemic (2014 and 2018) and after the peak of the pandemic (2023), each using identical designs, methods, and target populations to enable a consistent comparison. The study aimed to compare pre‐ and post‐COVID‐19 data to examine changes in adolescent internalizing and externalizing problems, deliberate self‐harm behavior, substance use, and help‐seeking behavior. We hypothesized that the prevalence of adolescent mental health problems and service utilization would be increased after the COVID‐19 pandemic.

## Materials and methods

### Participants and study procedure

This RoSa time‐trend study in Finland included three repeated cross‐sectional surveys conducted in spring 2014, 2018, and 2023, capturing data from both before and after the COVID‐19 pandemic. Participants were adolescents from Rovaniemi in Finnish Lapland (population ~ 60,000) and Salo in southern Finland (population ~ 52,000). Data collection was carried out in all secondary schools in these cities, excluding schools and classes for children with special needs.

**Figure 1:**
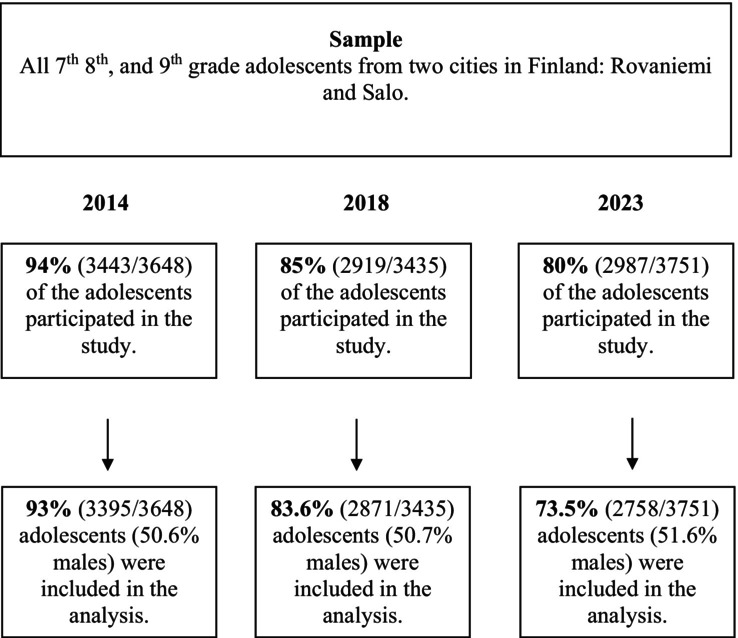
Flowchart of respondent inclusion

The study collected data from 7th to 9th graders in Finnish secondary schools, aged 13 to 16 years. All students present on the survey day were invited to participate, completing questionnaires during class and sealing their responses in envelopes. Absent students were given the opportunity to participate later. Across all 3 years, a total of 9,283 questionnaires were collected. We performed an initial screening to remove questionnaires with inappropriate responses (11 (0.3%) in 2014, 13 (0.4%) in 2018, and 42 (1.4%) in 2023), such as uniform response patterns, logically inconsistent or invalid answers, or a large amount of missing data. Additionally, students under 13, over 16, or with missing age or sex information were excluded, resulting in a final sample of 9,024 students for analysis (Figure 1). For all other variables, listwise deletion was applied given the minimal amount of missing data.

This study has been approved by the Ethics Committee of the University of Turku in all 3 years, 2014, 2018, and 2023. Participation was entirely voluntary. Parental consent was obtained by informing parents about the study in advance, giving them the option to decline their adolescent's participation. The study followed the ethical standards from the 1964 Declaration of Helsinki and its later amendments.

### Measures

In *demographic* section, we asked about their age, gender, city, school grade, family structure, and parental background. Family structure was categorized as living with two biological parents, one biological parent, or other. Parental background was based on country of birth and categorized as both parents born in Finland, one parent born in Finland, or neither parent born in Finland.

*Mental health* was assessed using the Strengths and Difficulties Questionnaire (SDQ) and one question about deliberate self‐harm behavior. The SDQ, translated and back‐translated from English to Finnish (Koskelainen, Sourander, & Vauras, 2001), includes 25 items across five scales: emotional symptoms, conduct problems, hyperactivity, peer problems, and prosocial behavior (Goodman, 1997; Goodman & Scott, 1999). Responses ranged from 0 (*not true*) to 2 (*certainly true*), with subscale scores ranging from 0 to 10, with higher scores indicating greater difficulties, except for prosocial behavior. Five items were phrased positively and scored in reverse. The total difficulties score, calculated by summing the scores from the first four subscales, ranged from 0 to 40. Additionally, perceived difficulties in emotions, concentration, behavior, or getting along with other people were asked, with the option of ‘No’ and ‘Yes (Minor, definite, or severe difficulties)’. Deliberate self‐harm behavior was assessed with one question, ‘Have you intentionally harmed yourself by cutting or burning your skin in the past 6 months?’ with response options of ‘no’, ‘once’, or ‘multiple times’.

*Substance use* was assessed through questions on alcohol consumption, drunkenness, smoking/nicotine use, and illegal drug use. Participants reported alcohol use and drunkenness frequency with options: ‘not at all’, ‘once a month or more often’, and ‘once a week or more often’. Smoking/nicotine use frequencies were categorized as: ‘not at all’, ‘not often’, ‘every week’, and ‘every day’. Illegal drug use was measured with options: ‘never’, ‘once’, ‘2–4 times’, and ‘5 or more times’.

*Help‐seeking behavior* was assessed by asking: ‘Within the past six months, have you at any point felt a need for outside help (someone outside your immediate family) with your problems, feelings, behavior, or emotional trouble?’ with three options: ‘No, I have not felt the need’, ‘I have considered getting outside help’, and ‘I have sought outside help’. Those selecting ‘I have sought outside help’ could specify the source, such as friends, relatives, or teachers.

### Statistical method

For background characteristics, frequencies and percentages were calculated for each year, and Pearson's Chi‐square test was used to compare the distributions. The outcomes were analyzed in two ways. First, categorical analyses used the SDQ total difficulties and subscores categorized by the 90th percentile based on the 2014 total sample (10th percentile for prosocial), as suggested by previous studies on both Finnish and broader European samples (Goodman, 1997; Koskelainen et al., 2001; Sourander, Helstelä, & Helenius, 1999). Mixed effects logistic regression with school‐wise random intercepts compared the likelihood of scores above these thresholds across years. Mixed effects multinomial logistic regression was used for overall difficulties, deliberate self‐harm behavior, and substance use, with ‘None’, ‘No’, and ‘Not at all’ as reference categories. For self‐perceived difficulties, the options ‘Definite’ and ‘Severe’ were combined. Second, SDQ subscales and total difficulties were analyzed as continuous variables using mixed effects linear regression. Mixed effects logistic regression was used to analyze the interaction effect of year and sex on the categorical outcomes. Significant interactions of sex were found for some outcomes; thus, the analyses were performed separately for females and males.

For all outcomes, odds ratios and mean differences were calculated for pairwise comparisons between the three time points: 2014 versus 2018, 2018 versus 2023, and 2014 versus 2023. To minimize the risk of false positive results due to multiple comparisons, the Bonferroni correction was applied by dividing the significance level of 0.05 by 3, resulting in a significance threshold of 0.0167 and 98.33% confidence intervals. A Type III test provided overall *p*‐values to determine if outcomes differed significantly across years, with *p*‐values <.05 considered significant. All results were adjusted for city, grade, family structure, and parents' background.

All statistical analyses were carried out with SAS software, version 9.4 for Windows. The full analysis code is openly available (Appendix S1).

## Results

### Participants sociodemographic

Significant changes were observed in city distribution and parental background between 2014 and 2023. The sample from Rovaniemi comprised 52.4% of the responses in 2014, 47.1% in 2018, and 51.1% in 2023 (*p* < .001). The percentage of participants with both parents born in Finland decreased significantly across the years (*p* < .001). No significant differences were observed in gender, school grade, or family structure (Table 1).

**Table 1:** Background characteristics of the participants in 2014, 2018, and 2023

Characteristics	2014	2018	2023	*p*‐Value
	(*N* = 3,395)	(*N* = 2,871)	(*N* = 2,758)	
Gender (%)^a^				0.706
Females	1,678 (49.4)	1,414 (49.3)	1,335 (48.4)	
Males	1,717 (50.6)	1,457 (50.7)	1,423 (51.6)	
School grade (%)^a^				0.708
7th graders	1,167 (34.4)	974 (34.0)	906 (32.9)	
8th graders	1,128 (33.2)	937 (32.7)	914 (33.2)	
9th graders	1,098 (32.4)	952 (33.3)	930 (33.8)	
Family structure (%)^a^				0.114
Two biological parents	2,369 (70.4)	2,067 (72.7)	1,928 (70.2)	
One biological parent	936 (27.8)	719 (25.3)	772 (28.1)	
Other	58 (1.7)	57 (2.0)	48 (1.7)	
Parents background (%)^a^				**<.001**
Both parents born in Finland	3,079 (91.7)	2,529 (89.1)	2,355 (86.7)	
One parent born in Finland	173 (5.2)	189 (6.7)	212 (7.8)	
Neither parent born in Finland	106 (3.2)	121 (4.3)	148 (5.5)	

Bold values indicate statistical significance in all tables.

^a^ Missing values for each variable: gender = 0 (0%); school grade = 18 (0.19%); family structure = 70 (0.75%); parents' background = 112 (1.2%).

### Changes in mental health

Significant sex interactions were found for SDQ total scores and all subscales, so results are presented separately for females and males (Table S1).

Table 2 shows results comparing 2023 to 2018. Females showed significant increases in SDQ total scores (OR 2.2, 98.33% CI 1.7–2.7), conduct problems (OR 1.7, 98.33% CI 1.1–2.7), emotional symptoms (OR 1.8, 98.33% CI 1.5–2.3), and hyperactivity (OR 2.8, 98.33% CI 2.2–3.6). Males showed a significant increase only in hyperactivity (OR 2.5, 98.33% CI 1.2–1.9). Females also reported a notable rise in ‘definite/severe’ perceived difficulties from 14.5% in 2018 to 28.3% in 2023 (OR 2.8, 98.33% CI 2.0–3.7), while in males, it increased from 7.5% to 10.7% (OR 1.5, 98.33% CI 1.0–2.1). No significant change was found in peer problems, prosocial behavior, or deliberate self‐harm behaviors for either sex during this period.

**Table 2:** Changes in SDQ total score and subscores, perceived difficulties, and deliberate self‐harm between three time points. Logistic regression analysis adjusted by city, grade, family structure, and parents' background

	2014	2018	2023	2023 vs. 2018	2023 vs. 2014	2018 vs. 2014	Overall year *p*‐value^c^
	%	%	%	OR (98.3% CI)^a^	OR (98.3% CI)^a^	OR (98.3% CI)^a^	
SDQ^c^ total score ≥cut‐off (18)^b^							
Females	16.9	17.0	30.6	2.1 (1.7–2.7)***	2.2 (1.7–2.7)***	1.0 (0.8–1.3)	**<0.001**
Males	8.0	9.0	11.5	1.3 (0.9–1.9)	1.6 (1.1–2.2)*	1.2 (0.8–1.7)	**0.011**
Conduct problems score ≥cut‐off (5)^b^							
Females	8.9	8.0	13.1	1.7 (1.1–2.7)**	1.4 (1.0–2.2)	0.8 (0.5–1.3)	0.008
Males	12.1	10.1	13.1	1.3 (1.0–1.7)	1.1 (0.8–1.4)	0.8 (0.6–1.1)	0.118
Emotional symptoms score ≥cut‐off (7)^b^							
Female	18.2	20.0	31.4	1.8 (1.5–2.3)***	2.1 (1.7–2.6)***	1.1 (0.9–1.4)	<.001
Males	2.3	4.3	3.2	0.8 (0.5–1.4)	1.5 (0.8–2.8)	1.9 (1.1–3.4)*	0.024
Hyperactivity symptoms score ≥cut‐off (6)^b^							
Female	18.1	18.2	38.1	2.8 (2.2–3.6)***	2.8 (2.2–3.5)***	1.0 (0.8–1.3)	<0.001
Males	13.0	12.8	18.5	1.5 (1.2–1.9)***	1.5 (1.2–1.9)***	1.0 (0.8–1.3)	<0.001
Peer problems score ≥cut‐off (4)^b^							
Females	18.9	21.4	24.8	1.2 (1.0–1.6)	1.4 (1.1–1.8)**	1.2 (0.9–1.5)	0.003
Males	17.4	19.7	21.6	1.1 (0.9–1.4)	1.3 (1.0–1.6)*	1.2 (0.9–1.5)	0.015
Prosocial behavior score ≤cut‐off (5)^b^							
Females	14.7	12.2	11.7	0.9 (0.7–1.3)	0.8 (0.6–1.0)	0.8 (0.6–1.1)	0.091
Males	30.1	28.3	29.1	1.1 (0.8–1.3)	1.0 (0.8–1.2)	0.9 (0.8–1.2)	0.733
Overall difficulties %							
Female							
Minor	34.3	39.6	38.5	1.3 (1.1–1.7)**	1.8 (1.5–2.2)***	1.3 (1.1–1.6)***	**<.001**
Definite/Severe	12.7	14.5	28.3	2.8 (2.0–3.7)***	3.6 (2.7–4.9)***	1.3 (1.0–1.8)	
Males							
Minor	30.9	29.5	31.6	1.2 (0.9–1.6)	1.2 (0.9–1.5)	1.0 (0.7–1.3)	**<.001**
Definite/Severe	5.3	7.5	10.7	1.5 (1.0–2.1)*	2.3 (1.6–3.2)***	1.5 (1.0–2.2)*	
Self‐harm %							
Females							
Once	12.1	15.2	13.3	0.9 (0.7–1.2)	1.2 (0.9–1.5)	1.3 (1.0–1.7)	**<.001**
Multiple times	6.9	9.1	12.2	1.4 (0.9–2.2)	2.0 (1.3–3.0)***	1.4 (0.9–2.1)	
Males							
Once	3.2	4.3	3.8	0.8 (0.5–1.3)	1.2 (0.7–1.9)	1.4 (0.9–2.3)	0.151
Multiple times	1.4	2.2	1.6	0.7 (0.4–1.4)	1.2 (0.6–2.5)	1.7 (0.8–3.3)	

Bold values indicate statistical significance in all tables.

^a^ Bonferroni correction; *p < .05, **p < .01, ***p < .001.

^b^ The cut‐off refers to the 90th percentile calculated based on 2014 scores, with the cut‐off value shown in parentheses.

^c^ *p*‐values are adjusted; *p < .05, **p < .01, ***p < .001.

**Figure 2:**
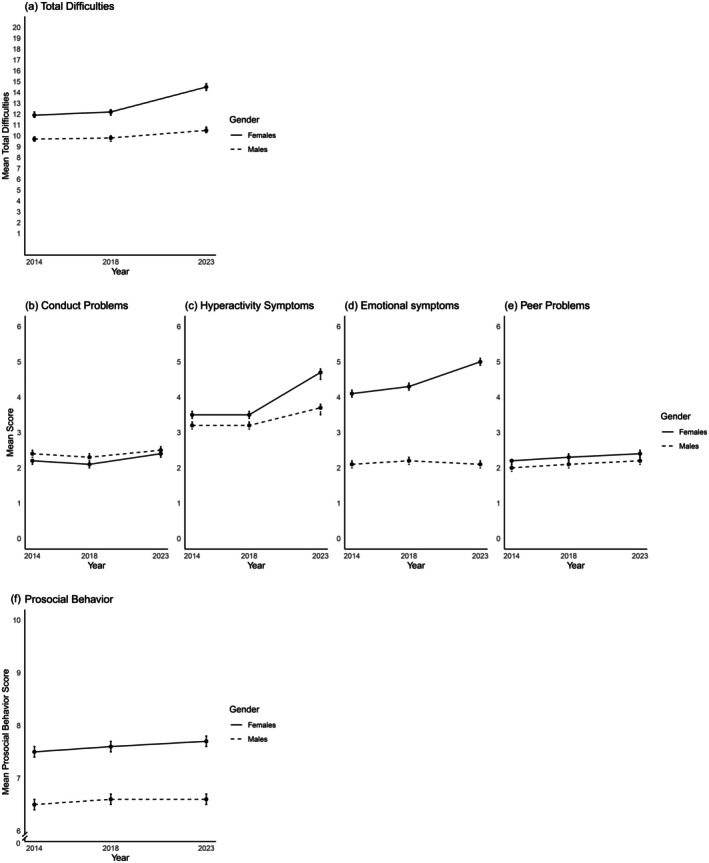
(A) The trend of SDQ total mean score by gender across survey years. (B–E) The trend of SDQ subscale scores by gender across survey years. (F) The trend of SDQ prosocial behavior mean score by gender across survey years

When comparing 2023–2014, similar trends were observed among females for total SDQ scores, emotional symptoms, and hyperactivity (all *p* < .001). Males showed an increase in SDQ total scores (OR 1.6, 98.33% CI 1.1–2.2) and hyperactivity (OR 1.5, 98.33% CI 1.2–1.9). In the pre‐COVID period (2014–2018), only minor increases were observed in emotional symptoms among males and in perceived minor difficulties for both sexes (Table 2). These patterns are further illustrated in Figure 2A–F, which describe changes in mean SDQ total and subscale scores separated by sex. Table S2 provides detailed changes in psychopathology by comparing mean values across different time points. The findings align with the categorical analysis, showing significant increases in psychopathological problems post‐COVID, with minimal changes between the two pre‐COVID assessments.

### Changes in deliberate self‐harm behavior

Deliberate self‐harm among females increased significantly only from 2014 to 2023, with the percentage of those reporting multiple self‐injury episodes rising from 6.9% to 12.2% (OR 2.0, 98.33% CI 1.3–3.0). Males showed no significant changes in deliberate self‐harm across the years (Table 2).

### Changes in substance use

Comparing 2018 to 2023, weekly smoking/nicotine use increased significantly among females (OR 2.7, 98.33% CI 1.5–4.9), while male alcohol use decreased (OR 0.7, 98.33% CI 0.5–0.9). No other significant changes in substance use were observed between 2014 and 2023 (Table 3).

**Table 3:** Changes in substance use between three time points. Logistic regression analysis adjusted by city, grade, family structure, and parents' background

	2014	2018	2023	2023 vs. 2018	2023 vs. 2014	2018 vs. 2014	Overall year *p*‐value^b^
	%	%	%	OR (98.3% CI)^a^	OR (98.3% CI)^a^	OR (98.3% CI)^a^	
Alcohol usage %							
Females							
Once a month or more	22.4	21.0	17.5	0.7 (0.5–1.0)	0.7 (0.5–0.9)*	0.9 (0.7–1.3)	.**036**
Once a week or more	1.4	1.3	0.9	0.7 (0.3–1.8)	0.6 (0.2–1.4)	0.8 (0.4–1.8)	
Males							
Once a month or more	23.2	20.9	15.1	0.7 (0.5–0.9)***	0.6 (0.5–0.7)***	0.9 (0.7–1.1)	**<.001**
Once a week or more	2.3	2.4	2.8	1.1 (0.6–2.2)	1.1 (0.6–2.1)	1.0 (0.5–1.9)	
Drunk %							
Females							
Once a month or more	16.2	14.7	12.9	0.8 (0.5–1.2)	0.7 (0.5–1.0)	0.9 (0.6–1.3)	.222
Once a week or more	0.7	0.6	0.5	0.8 (0.2–2.6)	0.7 (0.2–2.1)	0.9 (0.3–2.6)	
Males							
Once a month or more	16.0	14.5	11.5	0.8 (0.6–1.1)	0.7 (0.5–0.9)	0.9 (0.6–1.2)	.**043**
Once a week or more	1.4	1.2	1.8	1.5 (0.6–3.3)	1.2 (0.6–2.5)	0.8 (0.4–1.8)	
Smoke %							
Females							
Rarely	10.1	10.6	11.8	1.2 (0.9–1.6)	1.2 (0.9–1.7)	1.0 (0.8–1.4)	.**002**
Weekly	3.3	2.4	5.6	2.7 (1.5–4.9)***	1.8 (1.1–3.0)*	0.7 (0.4–1.2)	
Daily	5.7	4.7	7.0	1.5 (0.8–2.7)	1.1 (0.6–2.1)	0.8 (0.4–1.4)	
Males							
Rarely	12.3	10.5	10.5	0.9 (0.6–1.3)	0.8 (0.6–1.1)	0.8 (0.6–1.2)	.**014**
Weekly	4.7	3.1	4.1	1.3 (0.8–2.2)	0.8 (0.5–1.3)	0.6 (0.4–1.0)	
Daily	11.5	9.0	7.5	0.9 (0.6–1.5)	0.6 (0.4–1.0)*	0.7 (0.4–1.0)	
Illegal drugs %							
Females							
Rarely	1.8	2.1	2.4	1.2 (0.6–2.3)	1.2 (0.7–2.3)	1.0 (0.5–1.9)	.847
Weekly	0.5	1.0	1.0	1.0 (0.4–2.6)	1.5 (0.5–4.3)	1.5 (0.5–4.3)	
Daily	1.1	0.8	1.1	1.2 (0.4–3.9)	0.8 (0.3–2.2)	0.7 (0.2–0.9)	
Males							
Rarely	2.2	2.2	2.4	1.0 (0.5–2.2)	1.2 (0.5–2.5)	1.1 (0.5–2.4)	.098
Weekly	1.4	2.1	1.2	0.6 (0.3–1.2)	0.9 (0.4–2.0)	1.5 (0.8–3.0)	
Daily	1.1	0.8	1.9	2.2 (1.0–5.2)	1.8 (0.9–3.8)	0.8 (0.3–2.0)	

Bold values indicate statistical significance in all tables.

^a^ Bonferroni correction, *p < .05, **p < .01, ***p < .001.

^b^ *p*‐values are adjusted, *p < .05, **p < .01, ***p < .001.

### Changes in help‐seeking behavior

Among females, help‐seeking behavior increased significantly, with 22.2% having sought help and 25.2% considering by 2023 (sought help: OR 1.5, 98.33% CI 1.2–2.0; considered help: OR 2.2, 98.33% CI 1.7–2.9, compared to 2018). By contrast, no significant changes in help‐seeking were observed among males across years (Table 4).

**Table 4:** Changes in help‐seeking behavior between three time points. Logistic regression analysis adjusted by city, grade, family structure, and parents' background

	2014	2018	2023	2023 vs. 2018	2023 vs. 2014	2018 vs. 2014	Overall year
	%	%	%	OR (98.3% CI)^a^	OR (98.3% CI)^a^	OR (98.3% CI)^a^	*p*‐value^b^
Help‐seeking behavior %							
Females							
Have considered seeking help	15.8	14.5	25.2	2.2 (1.7–2.9)***	2.1 (1.7–2.7)***	0.9 (0.7–1.2)	**<.001**
Have sought help	16.1	18.6	22.2	1.5 (1.2–2.0)***	1.8 (1.4–2.4)***	1.2 (0.9–1.6)	
Males							
Have considered seeking help	5.4	5.9	6.5	1.0 (0.7–1.5)	1.2 (0.9–1.8)	1.2 (0.8–1.7)	.206
Have sought help	4.0	5.8	5.0	0.9 (0.6–1.4)	1.3 (0.8–2.1)	1.4 (0.9–2.3)	

Bold values indicate statistical significance in all tables.

^a^ Bonferroni correction, *p < .05, **p < .01, ***p < .001.

^b^ *p*‐values are adjusted, *p < .05, **p < .01, ***p < .001.

## Discussion

The current study reveals significant increases in mental health problems among Finnish adolescents before and after the COVID‐19 pandemic, particularly among females. Compared to prepandemic data from 2018, female adolescents in 2023 showed heightened rates of emotional symptoms, hyperactivity, conduct problems, and overall difficulties, while male adolescents demonstrated increases primarily in hyperactivity symptoms. Additionally, the frequency of deliberate self‐harm among females rose from 2014 to 2023. Substance use trends remained relatively stable, with an increase in weekly smoking among females as the only exception. These findings underscore a concerning postpandemic deterioration in adolescent mental health, especially among females, emphasizing the need for targeted mental health interventions and support.

This study offers several important contributions to the field by using repeated cross‐sectional surveys with the same design to comprehensively assess Finnish adolescent mental health before (2014, 2018) and after the pandemic (2023). Unlike studies focused solely on internalizing problems, this research takes a comprehensive approach, examining externalizing problems, deliberate self‐harm, substance use, and help‐seeking behavior. Multiple prepandemic reference points help isolate pandemic‐related changes, and the population‐based sample enhances the reliability and generalizability of the findings within the Finnish context.

Similar to our findings, previous studies have reported increases in internalizing disorders (Kiviruusu et al., 2024; Madigan, Racine, et al., 2023), eating disorders (Hartman‐Munick et al., 2022; Kiviruusu et al., 2024), and emergency visits for attempted suicide and self‐harm (Kiviruusu et al., 2024; Madigan, Korczak, et al., 2023) among adolescents after the onset of the COVID‐19 pandemic. Adolescents' sensitivity to global threats like the pandemic, climate crisis, and armed conflicts may contribute to these issues. Climate anxiety and worries, widely reported among adolescents, correlate with general anxiety and depression (Hickman et al., 2021; Lass‐Hennemann et al., 2024; Leonhardt, Granrud, Bonsaksen, & Lien, 2022; Sciberras & Fernando, 2022; Wu et al., 2023; Wullenkord, Tröger, Hamann, Loy, & Reese, 2021). Additionally, given Finland's proximity to Russia, the perceived threat of war may have been particularly impactful on Finnish adolescents (The Advisory Board for Defence Information [ABDI], 2024).

Consistent with our findings, emotional problems were discovered among females. A Finnish study reported increases in anxiety, depression, eating disorder symptoms, and suicidal behavior during and after the pandemic, while males showed no such increases (Kiviruusu et al., 2024). The gender difference highlights a critical area of concern that the pandemic's role in exacerbating pre‐existing gender disparities in mental health is consistent with prepandemic findings of greater internalizing symptoms among females (Deng et al., 2023; Kauhanen et al., 2023; Keyes & Platt, 2024). One contributing factor may be the heightened sensitivity of girls to some potential negative effects of social media exposure compared with boys (Keles, McCrae, & Grealish, 2020; Kelly, Zilanawala, Booker, & Sacker, 2018; Twenge, 2020). Some studies also suggested that females are more prone to climate anxiety (Heeren, Mouguiama‐Daouda, & Contreras, 2022; Wullenkord et al., 2021).

We also observed a rise in hyperactivity symptoms among both female and male adolescents, with a particularly sharp increase in females. This indicates growing trends of restlessness, impulsivity, and poor concentration, worsening after COVID‐19. These findings align with a Finnish population study reporting an 18.6% increase in ADHD diagnoses, especially among females aged 12–30 from 2019 to 2022 (Auro, Holopainen, Perola, Havulinna, & Raevuori, 2024). Changes in school, social, and learning environments during the pandemic may have contributed to this trend (Auro et al., 2024). While our study focused on hyperactivity, which differs from ADHD, the documented co‐occurrence of hyperactivity and anxiety may explain the parallel increase in emotional problems and hyperactivity symptoms (Jarrett & Ollendick, 2008).

It is concerning to note that deliberate self‐harm was already prevalent among adolescents before the pandemic, and this trend has only worsened. Deliberate self‐harm is common in the general adolescent population, particularly among females (Kiviruusu et al., 2024; Madigan, Korczak, et al., 2023; Monto, McRee, & Deryck, 2018). The higher prevalence among females aligns with results from the United States (Monto et al., 2018). Our findings align with global trends, showing 12‐month self‐harm rates of approximately 19.5% across high‐income and low‐ to middle‐income countries. Increases in adolescent hospitalizations for self‐harm injuries have been reported globally, including in Australia, Canada, the United States, and Europe, pre‐ and during COVID‐19 (Corrigan et al., 2022; Hiscock et al., 2022; Ougrin et al., 2022; Petruzzelli et al., 2022; Shankar et al., 2022; Steeg et al., 2021; Zhang, Davis, Finkelstein, & Rosenfield, 2022). The rise in deliberate self‐harm among females in this study may relate to increased psychopathology, while this study specifically examines general deliberate self‐harm behavior without considering underlying reasons, such as suicidal intentions. Furthermore, in this survey, we used a single item to assess deliberate self‐harm behavior, which may result in lower prevalence estimates compared to using a checklist (Aspeqvist, Andersson, Korhonen, Dahlström, & Zetterqvist, 2024). The item specifically referred to cutting or burning and may have excluded other common forms of self‐injurious behavior – such as self‐hitting, head‐banging, biting, or scratching oneself to cause pain – potentially leading to an underestimation of the overall prevalence of self‐harm.

Regarding substance use, our study found a relatively stable trend before and after the COVID‐19 pandemic, with decreases in alcohol use and drunkenness decreasing among females and males, aligning with findings from other Western countries. A systematic review of over 20 countries reported declines in adolescent substance use during the pandemic, including alcohol, cannabis, tobacco, and vaping (Layman et al., 2022), likely due to public health restrictions limiting access and socialization. However, while the trend of nicotine use among Finnish adolescents has previously been decreasing or stable (Mishina, Heinonen, Lempinen, & Sourander, 2024; Ollila & Ruokolainen, 2023), our findings suggest a reversal in this trend. The increase may be partly due to the rising popularity of new nicotine products, such as vaping and nicotine pouches. Finnish data have also reported increases in vaping since 2021, especially among adolescent females, and the use of nicotine pouches has reached the same level as the use of snus (Ollila & Ruokolainen, 2023). Considering the rising popularity of these new, alternative tobacco products, it is crucial to monitor this trend closely in the future (Speciale, Rao, Yang, & Nugent, 2023).

After the pandemic, there was a significant increase in females seeking mental health support, with nearly half actively considering or seeking help. The use of child and adolescent psychiatric services decreased early in the pandemic (Revet et al., 2022; Wan Mohd Yunus et al., 2022), but gradually increased later (Kalmin et al., 2023). Generally, women are more likely than men to seek help, and this difference may have been further accentuated by the pandemic (Westberg, Nyholm, Nygren, & Svedberg, 2022). Moreover, this study revealed a significant increase in emotional and conduct problems among females after the pandemic, potentially leading to increased help‐seeking behaviors.

There are several limitations to our study. First, the findings based on the Finnish sample may not be generalizable to other countries, but the populations of Rovaniemi and Salo were highly comparable with Finland's general population in terms of demographics (StatFin, 2023). Second, the study relies solely on self‐reports, which, though valuable for assessing adolescent mental health, could be strengthened by including input from parents or teachers. Third, the overall response rate is high, but it has slightly declined from 2014 to 2023. Nonparticipants may have had greater difficulties, potentially leading to an underestimation of problems in our sample (Kearney, 2008). However, since the study was conducted using similar methods each time, it is likely that absenteeism occurred similarly each year. Fourth, this study applied listwise deletion to handle missing data, which may introduce bias. However, given the very low proportion of missing data in our study, the impact on the results is likely minimal.

## Conclusion

This study provides valuable insights into adolescent mental health by analyzing the impact of COVID‐19 through three repeated cross‐sectional surveys. By examining internalizing and externalizing problems, deliberate self‐harm, substance use, and help‐seeking behavior, it offers a more integrated perspective. Our findings reveal a concerning trend of increased emotional symptoms and deliberate self‐harm among females, as well as a rise in hyperactivity among both genders. The findings highlight a decline in adolescent well‐being after the COVID‐19 pandemic, suggesting a prolonged trend and the need for continued attention to adolescent mental health, as well as the implementation of effective interventions, particularly for females.

Future research should continue monitoring these trends. The future RoSa study data collection using the same questionnaire and methodology is planned for 2026 and 2029. Additionally, it is important to explore the underlying mechanisms contributing to these mental health trends. Future research can address this issue through approaches such as qualitative research and longitudinal studies, which provide deeper insights into adolescents' experiences and the long‐term factors influencing their well‐being.

## Conflict of interests

The authors declare no competing interests.

## Funding

The European Research Council (ERC) under the European Union's Horizon 2020 research and innovation program (grant agreement No. 101020767; ERC Advanced, Andre Sourander); the Research Council of Finland (decision number: 345546); the Juho Vainio Foundation; the Academy of Finland Special funding for research on the COVID‐19 epidemic and the mitigation of its effects (decision number: 335690); and Nordforsk (Welfare among Children and Young People in the Post‐Pandemic Nordics, decision number 156858).

## Ethics

The Ethics Committee of the University of Turku approved the study (2014, 2018, and 2023). The study followed the ethical standards from the 1964 Declaration of Helsinki and its later amendments. Parental consent was obtained by informing parents about the study in advance, giving them the option to decline their adolescent's participation.

## Supporting information


**Appendix S1.** SAS codes.

**Table S1.** Descriptive statistics and changes in SDQ total and subscales across three time points (2014, 2018, and 2023). Mixed linear regression results are adjusted for city, grade, family structure, and parental background.

**Table S2.** Interaction effects of year and sex on adolescent mental health, self‐harm, substance use, and help‐seeking behaviors.

## Data Availability

The data that support the findings of this study are available from the author AS, upon reasonable request.

## References

[camh70040-bib-0001] Aspeqvist, E. , Andersson, H. , Korhonen, L. , Dahlström, Ö. , & Zetterqvist, M. (2024). Measurement and stratification of nonsuicidal self‐injury in adolescents. BMC Psychiatry, 24, 107. 38326791 10.1186/s12888-024-05535-3 PMC10848387

[camh70040-bib-0002] Auro, K. , Holopainen, I. , Perola, M. , Havulinna, A.S. , & Raevuori, A. (2024). Attention‐deficit/hyperactivity disorder diagnoses in Finland during the COVID‐19 pandemic. JAMA Network Open, 7, e2418204. 38935377 10.1001/jamanetworkopen.2024.18204 PMC11211961

[camh70040-bib-0004] Bower, M. , Smout, S. , Donohoe‐Bales, A. , O'Dean, S. , Teesson, L. , Boyle, J. , … & Teesson, M. (2023). A hidden pandemic? An umbrella review of global evidence on mental health in the time of COVID‐19. Frontiers in Psychiatry, 14, 1–19. 10.3389/fpsyt.2023.1107560 PMC10032377 36970258

[camh70040-bib-0005] Cheng, H. , Wang, D. , Wang, L. , Zou, H. , & Qu, Y. (2023). Global prevalence of self‐harm during the COVID‐19 pandemic: A systematic review and meta‐analysis. BMC Psychology, 11, 1–15. 37147683 10.1186/s40359-023-01181-8 PMC10160734

[camh70040-bib-0006] Collishaw, S. (2015). Annual Research Review: Secular trends in child and adolescent mental health. Journal of Child Psychology and Psychiatry, 56, 370–393. 10.1111/jcpp.12372 25496340

[camh70040-bib-0007] Corrigan, C. , Duke, G. , Millar, J. , Paul, E. , Butt, W. , Gordon, M. , … & Oberender, F. (2022). Admissions of children and adolescents with deliberate self‐harm to intensive care during the SARS‐CoV‐2 outbreak in Australia. JAMA Network Open, 5, e2211692. 35544133 10.1001/jamanetworkopen.2022.11692 PMC9096595

[camh70040-bib-0008] Deng, J. , Zhou, F. , Hou, W. , Heybati, K. , Lohit, S. , Abbas, U. , … & Heybati, S. (2023). Prevalence of mental health symptoms in children and adolescents during the COVID‐19 pandemic: A meta‐analysis. Annals of the New York Academy of Sciences, 1520, 53–73. 36537131 10.1111/nyas.14947 PMC9880764

[camh70040-bib-0009] Goodman, R. (1997). The strengths and difficulties questionnaire: A research note. Journal of Child Psychology and Psychiatry, and Allied Disciplines, 38, 581–586. 9255702 10.1111/j.1469-7610.1997.tb01545.x

[camh70040-bib-0010] Goodman, R. , & Scott, S. (1999). Comparing the strengths and difficulties questionnaire and the child behavior checklist: Is small beautiful? Journal of Abnormal Child Psychology, 27, 17–24. 10197403 10.1023/a:1022658222914

[camh70040-bib-0011] Gustafsson, J. , Lyyra, N. , Lahti, I.J. , Simonsen, N. , Lahti, H. , Kulmala, M. , … & Paakkari, L. (2023). Mental health profiles of Finnish adolescents before and after the peak of the COVID 19 pandemic. Child and Adolescent Psychiatry and Mental Health, 17, 54. 37120557 10.1186/s13034-023-00591-1 PMC10148589

[camh70040-bib-0012] Hafstad, G.S. , Sætren, S.S. , Wentzel‐Larsen, T. , & Augusti, E.M. (2022). Changes in adolescent mental and somatic health complaints throughout the COVID‐19 pandemic: A three‐wave prospective longitudinal study. Journal of Adolescent Health, 71, 406–413. 10.1016/j.jadohealth.2022.05.009 PMC9212438 35725540

[camh70040-bib-0013] Hartman‐Munick, S.M. , Lin, J.A. , Milliren, C.E. , Braverman, P.K. , Brigham, K.S. , Fisher, M.M. , … & Richmond, T.K. (2022). Association of the COVID‐19 pandemic with adolescent and young adult eating disorder care volume. JAMA Pediatrics, 176, 1225–1232. 36342721 10.1001/jamapediatrics.2022.4346 PMC9641596

[camh70040-bib-0014] Haslam, N. (2016). Concept creep: Psychology's expanding concepts of harm and pathology. Psychological Inquiry, 27, 1–17.

[camh70040-bib-0015] Heeren, A. , Mouguiama‐Daouda, C. , & Contreras, A. (2022). On climate anxiety and the threat it may pose to daily life functioning and adaptation: A study among European and African French‐speaking participants. Climatic Change, 173, 15. 35912274 10.1007/s10584-022-03402-2 PMC9326410

[camh70040-bib-0016] Hickman, C. , Marks, E. , Pihkala, P. , Clayton, S. , Lewandowski, E. , Mayall, E.E. , … & Van Susteren, L. (2021). Climate anxiety in children and young people and their beliefs about government responses to climate change: A global survey. The Lancet Planetary Health, 5, e863–e873. 34895496 10.1016/S2542-5196(21)00278-3

[camh70040-bib-0017] Hiscock, H. , Chu, W. , O'reilly, G. , Freed, G.L. , White, M. , Danchin, M. , & Craig, S. (2022). Association between COVID‐19 restrictions and emergency department presentations for paediatric mental health in Victoria, Australia. Australian Health Review, 46, 529–536. 35787299 10.1071/AH22015

[camh70040-bib-0018] Jarrett, M.A. , & Ollendick, T.H. (2008). A conceptual review of the comorbidity of attention‐deficit/hyperactivity disorder and anxiety: Implications for future research and practice. Clinical Psychology Review, 28, 1266–1280. 18571820 10.1016/j.cpr.2008.05.004

[camh70040-bib-0020] Kalmin, M.M. , Cantor, J.H. , Bravata, D.M. , Ho, P.C. , Whaley, C. , & McBain, R.K. (2023). Utilization and spending on mental health services among children and youths with commercial insurance. JAMA Network Open, 6, e2336979. 37787996 10.1001/jamanetworkopen.2023.36979 PMC10548294

[camh70040-bib-0021] Kauhanen, L. , Mohd, W. , Wan, A. , Yunus, M. , Lempinen, L. , Peltonen, K. , … & Sourander, A. (2023). A systematic review of the mental health changes of children and young people before and during the COVID 19 pandemic. European Child & Adolescent Psychiatry, 32, 995–1013. 35962147 10.1007/s00787-022-02060-0 PMC9373888

[camh70040-bib-0022] Kearney, C.A. (2008). School absenteeism and school refusal behavior in youth: A contemporary review. Clinical Psychology Review, 28, 451–471. 17720288 10.1016/j.cpr.2007.07.012

[camh70040-bib-0023] Keles, B. , McCrae, N. , & Grealish, A. (2020). A systematic review: The influence of social media on depression, anxiety and psychological distress in adolescents. International Journal of Adolescence and Youth, 25, 79–93.

[camh70040-bib-0024] Kelly, Y. , Zilanawala, A. , Booker, C. , & Sacker, A. (2018). Social media use and adolescent mental health: Findings from the UK millennium cohort study. EClinicalMedicine, 6, 59–68. 31193561 10.1016/j.eclinm.2018.12.005 PMC6537508

[camh70040-bib-0025] Keyes, K.M. , & Platt, J.M. (2024). Annual research review: Sex, gender, and internalizing conditions among adolescents in the 21st century – Trends, causes, consequences. Journal of Child Psychology and Psychiatry, and Allied Disciplines, 65, 384–407. 37458091 10.1111/jcpp.13864 PMC12341061

[camh70040-bib-0026] Kiviruusu, O. , Ranta, K. , Lindgren, M. , Haravuori, H. , Silén, Y. , Therman, S. , … & Suvisaari, J. (2024). Mental health after the COVID‐19 pandemic among Finnish youth: A repeated, cross‐sectional, population‐based study. The Lancet. Psychiatry, 11, 451–460. 38760112 10.1016/S2215-0366(24)00108-1

[camh70040-bib-0027] Koskelainen, M. , Sourander, A. , & Vauras, M. (2001). Self‐reported strengths and difficulties in a community sample of Finnish adolescents. European Child and Adolescent Psychiatry, 10, 180–185. 11596818 10.1007/s007870170024

[camh70040-bib-0028] Lass‐Hennemann, J. , Sopp, M.R. , Ruf, N. , Equit, M. , Schäfer, S.K. , Wirth, B.E. , & Michael, T. (2024). Generation climate crisis, COVID‐19, and Russia–Ukraine‐War: Global crises and mental health in adolescents. European Child and Adolescent Psychiatry, 33, 2203–2216. 37814081 10.1007/s00787-023-02300-x PMC11255088

[camh70040-bib-0029] Layman, H.M. , Thorisdottir, I.E. , Halldorsdottir, T. , Sigfusdottir, I.D. , Allegrante, J.P. , & Kristjansson, A.L. (2022). Substance use among youth during the COVID‐19 pandemic: A systematic review. Current Psychiatry Reports, 24, 307–324. 35476186 10.1007/s11920-022-01338-z PMC9043089

[camh70040-bib-0030] Leonhardt, M. , Granrud, M.D. , Bonsaksen, T. , & Lien, L. (2022). Associations between mental health, lifestyle factors and worries about climate change in Norwegian adolescents. International Journal of Environmental Research and Public Health, 19, 12826. 36232127 10.3390/ijerph191912826 PMC9565126

[camh70040-bib-0031] Madigan, S. , Korczak, D.J. , Vaillancourt, T. , Racine, N. , Hopkins, W.G. , Pador, P. , … & Neville, R.D. (2023). Comparison of paediatric emergency department visits for attempted suicide, self‐harm, and suicidal ideation before and during the COVID‐19 pandemic: A systematic review and meta‐analysis. The Lancet Psychiatry, 10, 342–351. 36907199 10.1016/S2215-0366(23)00036-6 PMC10097509

[camh70040-bib-0032] Madigan, S. , Racine, N. , Vaillancourt, T. , Korczak, D.J. , Hewitt, J.M.A. , Pador, P. , … & Neville, R.D. (2023). Changes in depression and anxiety among children and adolescents from before to during the COVID‐19 pandemic: A systematic review and meta‐analysis. JAMA Pediatrics, 177, 567–581. 37126337 10.1001/jamapediatrics.2023.0846 PMC10152379

[camh70040-bib-0033] Mishina, K. , Heinonen, E. , Lempinen, L. , & Sourander, A. (2024). Twenty‐year changes of adolescent mental health and substance use: A Finnish population‐based time‐trend study. European Child & Adolescent Psychiatry, 34, 685–694. 10.1007/s00787-024-02512-9 38985336 PMC11868224

[camh70040-bib-0034] Monto, M.A. , McRee, N. , & Deryck, F.S. (2018). Nonsuicidal self‐injury among a representative sample of US adolescents, 2015. American Journal of Public Health, 108, 1042–1048. 29927642 10.2105/AJPH.2018.304470 PMC6050840

[camh70040-bib-0035] Myhr, A. , Vesterbekkmo, R.K. , Samarawickrema, I. , & Sund, E.R. (2024). Trends in Norwegian adolescents' substance use between 2014 and 2022: Socioeconomic and gender differences. BMC Public Health, 24, 2482. 39267032 10.1186/s12889-024-19983-9 PMC11391704

[camh70040-bib-0036] Ollila, H. , & Ruokolainen, O. (2023). Tupakka‐ ja nikotiinituotteiden käyttö ja hankintatavat nuorilla oppilaitostyypeittäin 2017–2023. Tutkimuksesta tiiviisti 49/2023. Helsinki: Terveyden ja hyvinvoinnin laitos.

[camh70040-bib-0037] Orban, E. , Li, L.Y. , Gilbert, M. , Napp, A.K. , Kaman, A. , Topf, S. , … & Ravens‐Sieberer, U. (2024). Mental health and quality of life in children and adolescents during the COVID‐19 pandemic: A systematic review of longitudinal studies. Frontiers in Public Health, 11, 1–13. 10.3389/fpubh.2023.1275917 PMC10800626 38259801

[camh70040-bib-0038] Ougrin, D. , Wong, B.H.‐C. , Vaezinejad, M. , Plener, P.L. , Mehdi, T. , Romaniuk, L. , … & Landau, S. (2022). Pandemic‐related emergency psychiatric presentations for self‐harm of children and adolescents in 10 countries (PREP‐kids): A retrospective international cohort study. European Child and Adolescent Psychiatry, 31, 1–13. 10.1007/s00787-021-01741-6 PMC7937052 33677628

[camh70040-bib-0039] Panchal, U. , de Salazar Pablo, G. , Franco, M. , Moreno, C. , Parellada, M. , Arango, C. , & Fusar‐Poli, P. (2021). The impact of COVID‐19 lockdown on child and adolescent mental health: Systematic review. European Child & Adolescent Psychiatry, 32, 1151–1177. 34406494 10.1007/s00787-021-01856-w PMC8371430

[camh70040-bib-0040] Peng, B. , Reeves, K.K.L. , Lee, S.W.Y. , Chung, T.H.Y. , Hui, H.W.L. , Leung, A.H.L. , & Pang, J.C.Y. (2023). Physical, psychological, and behavioral problems among children and adolescents in countries with different economic statuses during the COVID‐19 pandemic: A systematic review and meta‐analysis. Frontiers in Pediatrics, 11, 1181186. 37342536 10.3389/fped.2023.1181186 PMC10277820

[camh70040-bib-0041] Petruzzelli, M.G. , Furente, F. , Colacicco, G. , Annecchini, F. , Margari, A. , Gabellone, A. , … & Matera, E. (2022). Implication of COVID‐19 pandemic on adolescent mental health: An analysis of the psychiatric counseling from the emergency room of an Italian University Hospital in the years 2019–2021. Journal of Clinical Medicine, 11, 6177. 36294498 10.3390/jcm11206177 PMC9604834

[camh70040-bib-0042] Ravens‐Sieberer, U. , Kaman, A. , Erhart, M. , Otto, C. , Devine, J. , Löffler, C. , … & Hölling, H. (2023). Quality of life and mental health in children and adolescents during the first year of the COVID‐19 pandemic: Results of a two‐wave nationwide population‐based study. European Child and Adolescent Psychiatry, 32, 575–588. 34636964 10.1007/s00787-021-01889-1 PMC8506100

[camh70040-bib-0043] Reiß, F. , Behn, S. , Erhart, M. , Strelow, L. , Kaman, A. , Ottová‐Jordan, V. , … & Ravens‐Sieberer, U. (2024). Subjective health and psychosomatic complaints of children and adolescents in Germany: Results of the HBSC study 2009/10 – 2022. Journal of Health Monitoring, 9, 7–22. 38559686 10.25646/11868 PMC10977472

[camh70040-bib-0044] Revet, A. , Hebebrand, J. , Anagnostopoulos, D. , Kehoe, L.A. , Banaschewski, T. , Bender, S. , … & Klauser, P. (2022). ESCAP CovCAP survey of heads of academic departments to assess the perceived initial (April/May 2020) impact of the COVID‐19 pandemic on child and adolescent psychiatry services. European Child & Adolescent Psychiatry, 31, 795–804. 33474653 10.1007/s00787-020-01699-x PMC7816838

[camh70040-bib-0045] Sayed, A.A. , El‐Gendy, A.A. , Aljohani, A.K. , Haddad, R.A. , Taher, O.H. , Senan, A.M. , … & Alqelaiti, B.A. (2024). The effects of COVID‐19 on the mental health of children and adolescents: A review. Cureus, 16, 8–13. 10.7759/cureus.56473 PMC11025694 38638779

[camh70040-bib-0046] Sciberras, E. , & Fernando, J.W. (2022). Climate change‐related worry among Australian adolescents: An eight‐year longitudinal study. Child and Adolescent Mental Health, 27, 22–29. 34766705 10.1111/camh.12521

[camh70040-bib-0047] Shankar, L.G. , Habich, M. , Rosenman, M. , Arzu, J. , Lales, G. , & Hoffmann, J.A. (2022). Mental health emergency department visits by children before and during the COVID‐19 pandemic. Academic Pediatrics, 22, 1127–1132. 35667622 10.1016/j.acap.2022.05.022 PMC9164513

[camh70040-bib-0048] Sourander, A. , Helstelä, L. , & Helenius, H. (1999). Parent‐adolescent agreement on emotional and behavioral problems. Social Psychiatry and Psychiatric Epidemiology, 34, 657–663. 10703276 10.1007/s001270050189

[camh70040-bib-0049] Speciale, Z. , Rao, S. , Yang, S. , & Nugent, K. (2023). An analysis of nicotine pouch use by middle school and high school students surveyed by the National Youth Tobacco Survey in 2021 and a review of the literature. Journal of Primary Care and Community Health, 14, 21501319231169994. 10.1177/21501319231169994 PMC10159243 37128171

[camh70040-bib-0050] StatFin . (2023). Key figures on population by region, 1990–2023. Available from: https://pxdata.stat.fi/PxWeb/pxweb/fi/StatFin/StatFin__vaerak/statfin_vaerak_pxt_11ra.px/. [last accessed 18 November 2024].

[camh70040-bib-0051] Steeg, S. , Bojanić, L. , Tilston, G. , Williams, R. , Jenkins, D.A. , Carr, M.J. , … & Webb, R.T. (2021). Temporal trends in primary care‐recorded self‐harm during and beyond the first year of the COVID‐19 pandemic: Time series analysis of electronic healthcare records for 2.8 million patients in the Greater Manchester Care Record. EClinicalMedicine, 41, 101175. 10.1016/j.eclinm.2021.101175 34746726 PMC8557994

[camh70040-bib-0052] The Advisory Board for Defence Information (ABDI) . (2024). Finns' opinions on foreign and security policy, national defence and security (The Advisory Board for Defence Information, Bulletins and Reports 2024:6). Ministry of Defence. Available from: http://urn.fi/URN:ISBN:978‐951‐663‐368‐1

[camh70040-bib-0053] Thorisdottir, I.E. , Agustsson, G. , Oskarsdottir, S.Y. , Kristjansson, A.L. , Asgeirsdottir, B.B. , Sigfusdottir, I.D. , … & Halldorsdottir, T. (2023). Effect of the COVID‐19 pandemic on adolescent mental health and substance use up to March, 2022, in Iceland: A repeated, cross‐sectional, population‐based study. The Lancet Child & Adolescent Health, 7, 347–357. 36913961 10.1016/S2352-4642(23)00022-6 PMC10005790

[camh70040-bib-0054] Twenge, J.M. (2020). Increases in depression, self‐harm, and suicide among U.S. adolescents after 2012 and links to technology use: Possible mechanisms. Psychiatric Research and Clinical Practice, 2, 19–25. 36101887 10.1176/appi.prcp.20190015 PMC9176070

[camh70040-bib-0055] Wan Mohd Yunus, W.M.A. , Kauhanen, L. , Sourander, A. , Brown, J.S.L. , Peltonen, K. , Mishina, K. , … & Gyllenberg, D. (2022). Registered psychiatric service use, self‐harm and suicides of children and young people aged 0–24 before and during the COVID‐19 pandemic: A systematic review. Child and Adolescent Psychiatry and Mental Health, 16, 15. 35216630 10.1186/s13034-022-00452-3 PMC8874300

[camh70040-bib-0056] Westberg, K.H. , Nyholm, M. , Nygren, J.M. , & Svedberg, P. (2022). Mental health problems among young people—A scoping review of help‐seeking. International Journal of Environmental Research and Public Health, 19, 1430. 35162452 10.3390/ijerph19031430 PMC8835517

[camh70040-bib-0057] Wiguna, T. , Minayati, K. , Kaligis, F. , Teh, S.D. , Sourander, A. , Dirjayanto, V.J. , … & Gilbert, S. (2024). The influence of screen time on behaviour and emotional problems among adolescents: A comparison study of the pre‐, peak, and post‐peak periods of COVID‐19. Heliyon, 10, e23325. 38163166 10.1016/j.heliyon.2023.e23325 PMC10755312

[camh70040-bib-0058] Wu, J. , Long, D. , Hafez, N. , Maloney, J. , Lim, Y. , & Samji, H. (2023). Development and validation of a youth climate anxiety scale for the youth development instrument survey. International Journal of Mental Health Nursing, 32, 1473–1483. 37605318 10.1111/inm.13201

[camh70040-bib-0059] Wullenkord, M.C. , Tröger, J. , Hamann, K.R.S. , Loy, L.S. , & Reese, G. (2021). Anxiety and climate change: A validation of the Climate Anxiety Scale in a German‐speaking quota sample and an investigation of psychological correlates. Climatic Change, 168, 20.

[camh70040-bib-0060] Zhang, E.W.J. , Davis, A. , Finkelstein, Y. , & Rosenfield, D. (2022). The effects of COVID‐19 on poisonings in the paediatric emergency department. Paediatrics and Child Health Canada, 27, S4–S8. 10.1093/pch/pxab100 PMC9126273 35620562

