# Supplementary material for: Self‐reported changes in adolescent mental health, deliberate self‐harm, substance use, and help‐seeking behavior before and after the COVID‐19 pandemic – A Finnish time‐trend study
Source: Child Adolesc Ment Health. 2025 Oct 8;31(1):13–22. doi: 10.1111/camh.70040 (PMC12832214; doi:10.1111/camh.70040)
Supplement: Supplementary file 1 — Appendix S1. SAS codes. [file CAMH-31-13-s002.docx]

**Self-reported Changes in Adolescent Mental Health, Deliberate Self-harm, Substance Use, and Help-seeking Behavior before and after COVID-19 pandemic – A Finnish time-trend study**

**Appendix S1: SAS codes**

**proc** **format**;

value problem **0**='<90th pctl' **1**='>=90th pctl';

value prosoc **0**='>10th pctl' **1**='<=10th pctl';

value familyf **1**='Two biological parents' **2**='Other';

value family3f **1**='Two biological parents' **2**='One biological parent' **3**='Other';

value backpar **1**='Both parents born in Finland' **2**='One parent born in Finland' **3**='Neither parent born in Finland';

value diff3f **0**='None' **1**='Minor' **2**='Definite/Severe';

**run**;

**data** data0;

set library.data_2014_2018_2023;

* Exclude if sex is missing or age is not between 13 and 17 ;

if sex in(**1**,**2**) and **13**<=age<=**17**;

* Perceived difficulties (SDQ item 26) had originally 4 categories. Combine two of them ;

if exp_diff_a in (**2**,**3**) then exp_diff_3cat=**2**;

else exp_diff_3cat=exp_diff_a;

*Prosocial behaviour (5 items): sdq1, sdq4, sdq9, sdq17, sdq20 / sconsid, sshares, scaring, skind, shelpout;

if n(sconsid,sshares,scaring,skind,shelpout) GE **3** then SDQ_prosoc=round(sum(sconsid,sshares,scaring,skind,shelpout)***5**/n(sconsid,sshares,scaring,skind,shelpout),**1**);

*Peer relationship problems (5 items): sdq6, sdq11, sdq14, sdq19, sdq23 / sloner, sfriend, spopular, sbullied, soldbest;

if sfriend ne **.** then sfrienx=**2**-sfriend;

if spopular ne **.** then spopulax=**2**-spopular;

if n(sloner,sfriend,spopular,sbullied,soldbest) GE **3** then SDQ_peer=round(sum(sloner,sfrienx,spopulax,sbullied,soldbest)***5**/n(sloner,sfriend,spopular,sbullied,soldbest),**1**);

*Hyperactivity/inattention (5 items): sdq2, sdq10, sdq15, sdq21, sdq25 / srestles, sfidgety, sdistrac, sreflect, sattends;

if sreflect ne **.** then sreflecx=**2**-sreflect;

if sattends ne **.** then sattendx=**2**-sattends;

if n(srestles,sfidgety,sdistrac,sreflect,sattends) GE **3** then SDQ_hyper=round(sum(srestles,sfidgety,sdistrac,sreflecx,sattendx)***5**/n(srestles,sfidgety,sdistrac,sreflect,sattends),**1**);

*Emotional symptoms (5 items): sdq3, sdq8, sdq13, sdq16, sdq24 / ssomatic, sworries, sunhappy, sclingy, safraid;

if n(ssomatic,sworries,sunhappy,sclingy,safraid) GE **3** then SDQ_emotion=round(sum(ssomatic,sworries,sunhappy,sclingy,safraid)***5**/n(ssomatic,sworries,sunhappy,sclingy,safraid),**1**);

*Conduct problems (5 items): sdq5, sdq7, sdq12, sdq18, sdq22 / stantrum, sobeys, sfights, slies, ssteals;

if sobeys ne **.** then sobeyx=**2**-sobeys;

if n(stantrum,sobeys,sfights,slies,ssteals) GE **3** then SDQ_conduct=round(sum(stantrum,sobeyx,sfights,slies,ssteals)***5**/n(stantrum,sobeys,sfights,slies,ssteals),**1**);

*A total difficulties score (based on 20 items);

if n(SDQ_emotion,SDQ_conduct,SDQ_hyper,SDQ_peer)=**4** then SDQ_ebdtot=SDQ_emotion+SDQ_conduct+SDQ_hyper+SDQ_peer;

label SDQ_emotion = "SDQ/Emotional symptoms"

SDQ_conduct = "SDQ/Conduct problems"

SDQ_hyper = "SDQ/Hyperactivity/inattention"

SDQ_peer = "SDQ/Peer problems"

SDQ_prosoc = "SDQ/Prosocial"

SDQ_ebdtot = "SDQ/Total difficulties";

label luokka_aste='School grade' perherak_3lk='Family structure'

exp_diff_3cat='Overall, do you think you have difficulties in any of the following areas: emotions, concentration, behaviour or getting along with other people?'

format perherak_3lk family3f. back_parents backpar. exp_diff_3cat diff3f. ;

**run**;

* calculate cutpoints of SDQ scales from 2014 data and create dichotomous variables based on them;

**proc** **means** data=data0 p10 p90 maxdec=**1**;

where vuosi=**2014** ;

var SDQ_ebdtot SDQ_conduct SDQ_emotion SDQ_hyper SDQ_peer SDQ_prosoc;

output out=sdqmeans p10= p90=/ autoname;

**run**;

**data** data1;

merge data0 sdqmeans;

by _type_;

drop _type_;

if sdq_ebdtot>=sdq_ebdtot_p90 then SDQ_tot_2cat=**1**;

else if sdq_ebdtot^=**.** then sdq_tot_2cat=**0**;

if sdq_conduct>=sdq_conduct_p90 then SDQ_conduct_2cat=**1**;

else if sdq_conduct^=**.** then SDQ_conduct_2cat=**0**;

if SDQ_emotion>=SDQ_emotion_p90 then SDQ_emotion_2cat=**1**;

else if SDQ_emotion^=**.** then SDQ_emotion_2cat=**0**;

if SDQ_hyper>=SDQ_hyper_p90 then SDQ_hyper_2cat=**1**;

else if SDQ_hyper^=**.** then SDQ_hyper_2cat=**0**;

if SDQ_peer>=SDQ_peer_p90 then SDQ_peer_2cat=**1**;

else if SDQ_peer^=**.** then SDQ_peer_2cat=**0**;

if **0**<=SDQ_prosoc<=SDQ_prosoc_p10 then SDQ_prosoc_2cat=**1**;

else if SDQ_prosoc^=**.** then SDQ_prosoc_2cat=**0**;

label SDQ_emotion_2cat = "SDQ/Emotional symptoms, 2cat, >=90% from 2014"

SDQ_conduct_2cat = "SDQ/Conduct problems, 2cat, >=90% from 2014"

SDQ_hyper_2cat = "SDQ/Hyperactivity/inattention, 2cat, >=90% from 2014"

SDQ_peer_2cat = "SDQ/Peer problems, 2cat, >=90% from 2014"

SDQ_prosoc_2cat = "SDQ/Prosocial, 2cat, <=10% from 2014"

SDQ_tot_2cat = "SDQ/Total difficulties, 2cat, >=90% from 2014"

;

format sdq_tot_2cat sdq_conduct_2cat sdq_emotion_2cat sdq_hyper_2cat sdq_peer_2cat bieds_2cat problem. SDQ_prosoc_2cat prosoc.;

**run**;

* Descriptive tables;

title color=blue "Distributions of demographic variables by year";

**proc** **freq** data=data1;

tables (city luokka_aste sex perherak_3lk back_parents)*vuosi /chisq;

**run**;

title color=blue "Distributions of outcomes by year and sex";

title2 color=darkblue "SDQ scores as categorical";

**proc** **tabulate** data=data1;

class vuosi sdq_tot_2cat sex;

table sex*sdq_tot_2cat,vuosi=''*(n pctn<sdq_tot_2cat>='%'*f=**8.1**);

**run**;

**proc** **tabulate** data=data1;

class vuosi sdq_conduct_2cat sex;

table sex*sdq_conduct_2cat,vuosi=''*(n pctn<sdq_conduct_2cat>='%'*f=**8.1**);

**run**;

**proc** **tabulate** data=data1;

class vuosi sdq_emotion_2cat sex;

table sex*sdq_emotion_2cat,vuosi=''*(n pctn<sdq_emotion_2cat>='%'*f=**8.1**);

**run**;

**proc** **tabulate** data=data1;

class vuosi sdq_hyper_2cat sex;

table sex*sdq_hyper_2cat,vuosi=''*(n pctn<sdq_hyper_2cat>='%'*f=**8.1**);

**run**;

**proc** **tabulate** data=data1;

class vuosi sdq_peer_2cat sex;

table sex*sdq_peer_2cat,vuosi=''*(n pctn<sdq_peer_2cat>='%'*f=**8.1**);

**run**;

**proc** **tabulate** data=data1;

class vuosi sdq_prosoc_2cat sex;

table sex*sdq_prosoc_2cat,vuosi=''*(n pctn<sdq_prosoc_2cat>='%'*f=**8.1**);

**run**;

title2 color=darkblue "SDQ scores as continuous";

**proc** **tabulate** data=data1;

class vuosi sex;

classlev sex/;

var SDQ_ebdtot SDQ_conduct SDQ_emotion SDQ_hyper SDQ_peer SDQ_prosoc;

table (SDQ_ebdtot SDQ_conduct SDQ_emotion SDQ_hyper SDQ_peer SDQ_prosoc)*sex='',vuosi=''*(mean std='SD')*f=**8.1**/;

**run**;

title2 color=darkblue "Difficulties, self-harm, seeking help and substance use";

**proc** **tabulate** data=data1;

class vuosi exp_diff_3cat sex;

table sex*exp_diff_3cat,vuosi=''*(n pctn<exp_diff_3cat>='%'*f=**8.1**);

**run**;

**proc** **tabulate** data=data1;

class vuosi Sui_A sex;

table sex*Sui_A,vuosi=''*(n pctn<Sui_A>='%'*f=**8.1**);

**run**;

**proc** **tabulate** data=data1;

class vuosi help_a sex;

table sex*help_a,vuosi=''*(n pctn<help_a>='%'*f=**8.1**);

**run**;

**proc** **tabulate** data=data1;

class vuosi alcohol sex;

table sex*alcohol,vuosi=''*(n pctn<alcohol>='%'*f=**8.1**);

**run**;

**proc** **tabulate** data=data1;

class vuosi drunkenness sex;

table sex*drunkenness,vuosi=''*(n pctn<drunkenness>='%'*f=**8.1**);

**run**;

**proc** **tabulate** data=data1;

class vuosi smoking sex;

table sex*smoking,vuosi=''*(n pctn<smoking>='%'*f=**8.1**);

**run**;

**proc** **tabulate** data=data1;

class vuosi drugs sex;

table sex*drugs,vuosi=''*(n pctn<drugs>='%'*f=**8.1**);

**run**;

title color=indigo "Categorical outcomes";

title2 color=darkblue "Interaction of year and sex";

**%macro** glmix_ia(resp, multi=**0**);

ods select tests3 ;

proc glimmix data=data1 order=internal method=laplace;

class &resp vuosi sex/ref=first ;

%if &multi=**1** %then %do;

model &resp(ref=first) = vuosi|sex/link=glogit dist=multinomial solution ;

random intercept /subject=school group=&resp type=chol ;

%end;

%else %do;

model &resp(ref=first) = vuosi|sex/link=logit dist=binary solution;

random intercept /subject=school ;

%end;

run;

**%mend** glmix_ia;

ods text="^S={fontsize=12pt color=blue just=c}SDQ Total difficulties score cutpoint 90th pctl (2014)";

%***glmix_ia***(sdq_tot_2cat)

ods text="^S={fontsize=12pt color=blue just=c}^n SDQ Conduct score cutpoint 90th pctl (2014)";

%***glmix_ia***(sdq_conduct_2cat)

ods text="^S={fontsize=12pt color=blue just=c}^n SDQ Emotional score cutpoint 90th pctl (2014)";

%***glmix_ia***(sdq_emotion_2cat)

ods text="^S={fontsize=12pt color=blue just=c}^n SDQ Hyperactivity score cutpoint 90th pctl (2014)";

%***glmix_ia***(sdq_hyper_2cat)

ods text="^S={fontsize=12pt color=blue just=c}^n SDQ Peer score cutpoint 90th pctl (2014)";

%***glmix_ia***(sdq_peer_2cat)

ods text="^S={fontsize=12pt color=blue just=c}^n SDQ Prosocial score cutpoint 90th pctl (2014)";

%***glmix_ia***(sdq_prosoc_2cat)

ods text="^S={fontsize=12pt color=blue just=c}Perceived difficulties (3 categories)";

%***glmix_ia***(exp_diff_3cat,multi=**1**);

ods text="^S={fontsize=12pt color=blue just=c}^n Self-harm";

%***glmix_ia***(sui_a,multi=**1**);

ods text="^S={fontsize=12pt color=blue just=c}^n Help-seeking";

%***glmix_ia***(help_a,multi=**1**);

ods text="^S={fontsize=12pt color=blue just=c}^n Drinking alcohol";

%***glmix_ia***(alcohol,multi=**1**);

ods text="^S={fontsize=12pt color=blue just=c}^n Getting drunk";

%***glmix_ia***(drunkenness,multi=**1**);

ods text="^S={fontsize=12pt color=blue just=c}^n Smoking";

%***glmix_ia***(smoking,multi=**1**);

ods text="^S={fontsize=12pt color=blue just=c}^n Illegal drugs";

%***glmix_ia***(drugs,multi=**1**);

**** Unadjusted mixed log. regression with Bonferroni corrections (alpha=0.05/3) by sex ****;

**%macro** glimmix_SDQ_bonf(resp, covs,multi=**0**);

ods select ResponseProfile tests3 dimensions covparms estimates;

proc glimmix data=data1 order=internal method=laplace;

class vuosi &covs &resp/ref=first ;

%if &multi=**1** %then %do;

model &resp(ref=first) = vuosi &covs/link=glogit dist=multinomial solution;

random intercept /subject=school group=&resp type=chol ;

*nloptions absgconv=0.0004;

%end;

%else %do;

model &resp(ref=first) = vuosi &covs/link=logit dist=binary solution;

random intercept /subject=school ;

%end;

estimate '2023 vs. 2014' vuosi **0** **1** -**1**,

'2023 vs. 2018' vuosi -**1** **1** **0**,

'2018 vs. 2014' vuosi **1** **0** -**1**/cl exp bycategory alpha=**0.01667** ;

by sex;

run;

**%mend** glimmix_SDQ_bonf;

**proc** **sort** data=data1;by sex;**run**;

title color=blue "Unadjusted mixed logistic regression";

%***glimmix_SDQ_bonf***(sdq_tot_2cat)

%***glimmix_SDQ_bonf***(sdq_conduct_2cat)

%***glimmix_SDQ_bonf***(sdq_emotion_2cat)

%***glimmix_SDQ_bonf***(sdq_hyper_2cat)

%***glimmix_SDQ_bonf***(sdq_peer_2cat)

%***glimmix_SDQ_bonf***(sdq_prosoc_2cat)

%***glimmix_SDQ_bonf***(exp_diff_3cat,multi=**1**);

%***glimmix_SDQ_bonf***(sui_a,multi=**1**);

%***glimmix_SDQ_bonf***(help_a,multi=**1**);

%***glimmix_SDQ_bonf***(alcohol,multi=**1**);

%***glimmix_SDQ_bonf***(drunkenness,multi=**1**);

%***glimmix_SDQ_bonf***(smoking,multi=**1**);

%***glimmix_SDQ_bonf***(drugs,multi=**1**);

title color=indigo "Continuous SDQ scales";

title2 color=darkblue "Interaction of year and sex";

**%macro** lmix_ia_catyear(resp);

ods select tests3;

proc mixed data=data1;

class vuosi sex/ ref=first;

model &resp = vuosi|sex ;

random intercept/subject=school;

run; quit;

**%mend** lmix_ia_catyear;

%***lmix_ia_catyear***(SDQ_ebdtot)

%***lmix_ia_catyear***(SDQ_hyper)

%***lmix_ia_catyear***(SDQ_emotion)

%***lmix_ia_catyear***(SDQ_conduct)

%***lmix_ia_catyear***(SDQ_peer)

%***lmix_ia_catyear***(SDQ_prosoc)

**** Unadjusted mixed lin. regression with Bonferroni corrections (alpha=0.05/3) ****;

**%macro** linmix_bonf(resp, covs);

ods exclude classlevels convergenceStatus fitstatistics iterHistory optInfo ;

proc mixed data=data1 order=internal;

class vuosi &covs/ref=first ;

model &resp = vuosi &covs/;

lsmeans vuosi/diff=all;

random intercept /subject=school ;

estimate '2023 vs. 2014' vuosi **0** **1** -**1**/cl alpha=**0.01667** ;

estimate '2023 vs. 2018' vuosi -**1** **1** **0**/cl alpha=**0.01667** ;

estimate '2018 vs. 2014' vuosi **1** **0** -**1**/cl alpha=**0.01667** ;

by sex;

run;

**%mend** linmix_bonf;

%***linmix_bonf***(SDQ_ebdtot);

%***linmix_bonf***(SDQ_conduct);

%***linmix_bonf***(SDQ_emotion);

%***linmix_bonf***(SDQ_hyper);

%***linmix_bonf***(SDQ_peer);

%***linmix_bonf***(SDQ_prosoc);

title color=blue "Mixed logistic regression adjusted by city, grade, family structure and parents' background";

title2 color=bib "Total difficulties";

%***glimmix_SDQ_bonf***(sdq_tot_2cat,city luokka_aste perherak_3lk back_parents)

title2 color=bib "Conduct problems";

%***glimmix_SDQ_bonf***(sdq_conduct_2cat,city luokka_aste perherak_3lk back_parents)

title2 color=bib "Emotional symptoms";

%***glimmix_SDQ_bonf***(sdq_emotion_2cat,city luokka_aste perherak_3lk back_parents)

title2 color=bib "Hyperactivity";

%***glimmix_SDQ_bonf***(sdq_hyper_2cat,city luokka_aste perherak_3lk back_parents)

title2 color=bib "Peer problems";

%***glimmix_SDQ_bonf***(sdq_peer_2cat,city luokka_aste perherak_3lk back_parents)

title2 color=bib "Prosocial behavior";

%***glimmix_SDQ_bonf***(sdq_prosoc_2cat,city luokka_aste perherak_3lk back_parents)

title2 color=bib "Perceived difficulties";

%***glimmix_SDQ_bonf***(exp_diff_3cat,city luokka_aste perherak_3lk back_parents,multi=**1**);

title2 color=bib "Self-harm";

%***glimmix_SDQ_bonf***(sui_a,city luokka_aste perherak_3lk back_parents,multi=**1**);

title2 color=bib "Seeking help";

%***glimmix_SDQ_bonf***(help_a,city luokka_aste perherak_3lk back_parents,multi=**1**);

title2 color=bib "Drinking alcohol";

%***glimmix_SDQ_bonf***(alcohol,city luokka_aste perherak_3lk back_parents,multi=**1**);

title2 color=bib "Getting drunk";

%***glimmix_SDQ_bonf***(drunkenness,city luokka_aste perherak_3lk back_parents,multi=**1**);

title2 color=bib "Smoking";

%***glimmix_SDQ_bonf***(smoking,city luokka_aste perherak_3lk back_parents,multi=**1**);

title2 color=bib "Illegal drugs";

%***glimmix_SDQ_bonf***(drugs,city luokka_aste perherak_3lk back_parents,multi=**1**);

title color=darkblue "Mixed linear regression results adjusted by city, grade, family structure and parents' background (with Bonferroni correction)";

%***linmix_bonf***(SDQ_ebdtot,city luokka_aste perherak_3lk back_parents);

%***linmix_bonf***(SDQ_conduct,city luokka_aste perherak_3lk back_parents);

%***linmix_bonf***(SDQ_emotion,city luokka_aste perherak_3lk back_parents);

%***linmix_bonf***(SDQ_hyper,city luokka_aste perherak_3lk back_parents);

%***linmix_bonf***(SDQ_peer,city luokka_aste perherak_3lk back_parents);

%***linmix_bonf***(SDQ_prosoc,city luokka_aste perherak_3lk back_parents);
