# Supplementary material for: Self‐reported changes in adolescent mental health, deliberate self‐harm, substance use, and help‐seeking behavior before and after the COVID‐19 pandemic – A Finnish time‐trend study
Source: Child Adolesc Ment Health. 2025 Oct 8;31(1):13–22. doi: 10.1111/camh.70040 (PMC12832214; doi:10.1111/camh.70040)
Supplement: Supplementary file 2 — Table S1. Descriptive statistics and changes in SDQ total and subscales across three time points (2014, 2018, and 2023). Mixed linear regression results are adjusted for city, grade, family structure, and parental background. [file CAMH-31-13-s003.docx]

**Supplementary table 1.** Descriptive statistics and changes in SDQ total and subscales across three-time points (2014, 2018, and 2023). Mixed linear regression results are adjusted for city, grade, family structure, and parental background.

|  |  | Mean (SD) | | | Estimate (98.33% CI) | | | P value |
| --- | --- | --- | --- | --- | --- | --- | --- | --- |
|  |  | 2014 | 2018 | 2023 | 2023 vs. 2018 | 2023 vs. 2014 | 2018 vs. 2014 |  |
| SDQ total | Female | 11.9 (5.7) | 12.2 (5.4) | 14.5 (6.0) | 2.4 (1.6 - 3.2)*** | 2.6 (1.8 - 3.3)*** | 0.2 (-0.6 - 0.9) | ******* |
|  | Male | 9.7 (4.9) | 9.8 (5.2) | 10.5 (5.3) | 0.7 (0.3 - 1.2)*** | 0.8 (0.4 - 1.3)*** | 0.1 (-0.4 - 0.5) | ******* |
| Conduct problems | Female | 2.2 (1.6) | 2.1 (1.6) | 2.4 (1.8) | 0.3 (0.1 - 0.6)** | 0.2 (0.0 - 0.5)* | -0.1 (-0.3 - 0.1) | ****** |
|  | Male | 2.4 (1.6) | 2.3 (1.6) | 2.5 (1.7) | 0.2 (0.0 - 0.4)* | 0.1 (-0.1 - 0.3) | -0.1 (-0.3 - 0.1) | ***** |
| Emotional | Female | 4.1 (2.4) | 4.3 (2.4) | 5.0 (2.5) | 0.7 (0.4 - 1.0)*** | 0.9 (0.6 - 1.2)*** | 0.2 (-0.1 - 0.5) | ******* |
|  | Male | 2.1 (1.9) | 2.2 (2.0) | 2.1 (1.9) | -0.0 (-0.2 - 0.2) | 0.1 (-0.1 - 0.3) | 0.1 (-0.1 - 0.3) | ns |
| Hyperactivity | Female | 3.5 (2.2) | 3.5 (2.2) | 4.7 (2.6) | 1.2 (0.9 - 1.4)*** | 1.1 (0.9 - 1.4)*** | -0.0 (-0.3 - 0.2) | ******* |
|  | Male | 3.2 (2.0) | 3.2 (2.0) | 3.7 (2.2) | 0.4 (0.2 - 0.6)*** | 0.4 (0.3 - 0.6)*** | 0.1 (-0.1 - 0.2) | ******* |
| Peer problems | Female | 2.2 (1.7) | 2.3 (1.8) | 2.4 (1.8) | 0.1 (-0.0 - 0.3) | 0.3 (0.1 - 0.4)** | 0.1 (-0.1 - 0.3) | ****** |
|  | Male | 2.0 (1.7) | 2.1 (1.8) | 2.2 (1.9) | 0.2 (0.0 - 0.4)* | 0.2 (0.1 - 0.4)** | 0.0 (-0.1 - 0.2) | ****** |
| Procosical | Female | 7.5 (1.8) | 7.6 (1.7) | 7.7 (1.7) | 0.1 (-0.1 - 0.3) | 0.2 (0.0 - 0.4)* | 0.1 (-0.1 - 0.3) | ***** |
|  | Male | 6.5 (1.9) | 6.6 (2.0) | 6.6 (2.1) | -0.0 (-0.3 - 0.2) | 0.1 (-0.2 - 0.3) | 0.1 (-0.1 - 0.4) | ns |

^a^Bonferroni correction

∗ p < .05. ∗∗ p < .01. ∗∗∗ p < .001.
