# Supplementary material for: Self‐reported changes in adolescent mental health, deliberate self‐harm, substance use, and help‐seeking behavior before and after the COVID‐19 pandemic – A Finnish time‐trend study
Source: Child Adolesc Ment Health. 2025 Oct 8;31(1):13–22. doi: 10.1111/camh.70040 (PMC12832214; doi:10.1111/camh.70040)
Supplement: Supplementary file 3 — Table S2. Interaction effects of year and sex on adolescent mental health, self‐harm, substance use, and help‐seeking behaviors. [file CAMH-31-13-s001.docx]

**Supplementary table 2.** Interaction effects of year and sex on adolescent mental health, self-harm, substance use, and help-seeking behaviors.

|  | **Year** | **Sex** | **Year*Sex** |
| --- | --- | --- | --- |
|  | **F** | **F** | **F** |
| Mental Health |  |  |  |
| Total Difficulties | 29.32*** | 172.58*** | 4.35** |
| Emotional Symptoms | 10.31*** | 534.04*** | 8.59*** |
| Hyperactivity | 78.16*** | 116.32*** | 13.48*** |
| Conduct Problems | 8.84*** | 6.66* | 1.34 |
| Peer Problems | 10.76*** | 5.58* | 0.23 |
| Prosocial Scores | 2.78 | 237.26*** | 1.14 |
| Self-Perceived Well-Being | 31.70*** | 128.72*** | 4.60** |
| Self-Harm Behavior | 4.46* | 7.93*** | 0.69 |
| Substance Use |  |  |  |
| Smoking | 2.81* | 5.79** | 2.37* |
| Alcohol Use | 2.00 | 6.21** | 0.33 |
| Drunkenness | 1.54 | 2.68* | 0.48 |
| Illegal Drug Use | 2.00 | 6.21** | 0.33 |
| Help-Seeking | 11.22*** | 348.85*** | 5.30*** |

∗ p < .05. ∗∗ p < .01. ∗∗∗ p < .001.
